# Non-alcoholic fatty liver disease—A pilot study investigating early inflammatory and fibrotic biomarkers of NAFLD with alcoholic liver disease

**DOI:** 10.3389/fphys.2022.963513

**Published:** 2022-12-15

**Authors:** Joanne Watt, Mary Jo Kurth, Cherith N. Reid, John V. Lamont, Peter Fitzgerald, Mark W. Ruddock

**Affiliations:** Randox Laboratories Ltd., Randox Science Park, Antrim, United Kingdom

**Keywords:** simple steatosis, NAFLD/NASH, ALD, IL-6, IL-8, TNFα, PIIINP, ST2/IL-33R

## Abstract

**Introduction**: Non-alcoholic fatty liver disease (NAFLD) is a condition where excess fat accumulates in the liver (hepatic steatosis) and there is no history of alcohol abuse or other secondary causes of chronic liver disease. NAFLD is a very common disorder, occurring in 25% of the global population. NAFLD is now the most common chronic liver disorder in Western countries. Liver biopsy is the gold standard for NAFLD diagnosis and staging; however, this is invasive, costly and not without risk. Biomarkers that could diagnose and stage disease would reduce the need for biopsy and allow stratification of patients at risk of progression to non-alcoholic steatohepatitis (NASH).

**Methods**: One hundred and thirty-five patients were involved in the study [N = 135: n = 34 controls; n = 26 simple steatosis; n = 61 NAFLD/NASH, and n = 14 alcoholic liver disease (ALD)]. Clinically diagnosed (ICD-10) patient serum samples were obtained from Discovery Life Sciences (US) along with clinical history. Samples were run in duplicate using high-sensitivity cytokine array I, immunoassays and ELISAs. In total, n = 20 individual biomarkers were investigated in this pilot study.

**Results**: Thirteen/20 (65%) biomarkers were identified as significantly different between groups; IFNγ, EGF, IL-1β, IL-6, IL-8, IL-10, TNFα, FABP-1, PIIINP, ST2/IL-33R, albumin, AST and ALT. Five/20 (25%) biomarker candidates were identified for further investigation; namely, three biomarkers of inflammation, IL-6, IL-8, and TNFα, and two biomarkers of fibrosis, PIIINP and ST2/IL-33R.

**Discussion**: Single biomarkers are unlikely to be diagnostic or predictive at staging NAFLD due to the complex heterogeneity of the disease. However, biomarker combinations may help stratify risk and stage disease where patients are averse to biopsy. Further studies comparing the 5 biomarkers identified in this study with current diagnostic tests and fibrotic deposition in liver tissue are warranted.

## Introduction

Non-alcoholic fatty liver disease (NAFLD) is a generic term for a spectrum of conditions caused by a build-up of fat in the liver (Smith and Adams, 2011). Individuals who are overweight or obese are at greatest risk (Lu et al., 2018). Fatty liver or NAFLD usually has no symptoms. However, if unchecked, NAFLD can lead to serious liver damage, including cirrhosis. Consequently, longitudinal follow-up studies have demonstrated that NAFLD is not benign as previously envisaged (De and Duseja, 2020). NAFLD develops in four stages: simple fatty liver (steatosis), non-alcoholic steatohepatitis (NASH), fibrosis, and cirrhosis (this damage is permanent and can lead to liver failure and/or liver cancer) (Kudaravalli and John, 2022). NAFLD affects an estimated 25% of the global population (Muhammad, 2019; Westfall et al., 2020) The disease affects all populations, all age groups and is the most common liver disorder in western industrialised countries (Tomic et al., 2018). Liver biopsy is the gold standard for NAFLD diagnosis and staging of the disease (Nalbantoglu and Brunt, 2014). However, this is an invasive and costly procedure, not without risk to the patient (Isabela Andronescu et al., 2018). Ultrasonogram (USG) imaging can show fat accumulation in the liver however, USG cannot show the extent of the inflammation or fibrosis (Gomercić et al., 2009). One in three people in the United Kingdom is estimated to be affected by NAFLD, which is closely related to the increased frequency in overweight and obese individuals (Non-alcoholic fatty liver disease, 2022). One of the pathologic hallmarks of obesity is macrophage infiltration of adipose tissue, a source of multipotent adult stem cells. Stem cell growth factor-beta (SCGF-B) activates both granulocyte and macrophage colony-stimulating factor, and SCGF-B, in concert with IL-6, have been implicated in the pathogenesis of obesity-related NAFLD in males (Tarantino et al., 2020). Metabolic-associated liver disease (MAFLD), which is commonly employed to describe NAFLD individuals, requires evidence of hepatic steatosis, obesity, type-2 diabetes and metabolic dysregulation (e.g., large waist circumference, inflammation, high blood pressure, decreased HDL-cholesterol, increased triglycerides, and insulin resistance) (Huang et al., 2021).

The liver helps the body maintain homeostasis by playing an essential role in lipid (fat) metabolism, i.e., fat is broken down for energy; excess glucose is converted into fat for storage. Healthy liver cells should contain little or no fat (<5%). However, excessive fat accumulation in the liver causes inflammation which damages liver cells that can stimulate the progression from steatosis to non-alcoholic steatohepatitis (NASH) (Marra and Lotersztajn, 2013). The pathogenesis of NAFLD is the subject of extensive research (Ma and Li, 2006; Magee et al., 2016; Parthasarathy et al., 2020). Blood-based tests (i.e., Fibrosis-4 (FIB-4) and enhanced liver fibrosis (ELF)) can be used to detect advanced fibrosis (Shah et al., 2009; López et al., 2017). However, these non-invasive tests, as alternatives to biopsy, have several limitations, such as variability, accuracy, and sampling error, which can lead to underreporting when compared to the gold standard (Srivastava et al., 2019). Furthermore, abnormalities are rarely investigated to the extent recommended by national guidelines (Macpherson et al., 2020).

The primary goal of this pilot study was to potentially identify 1) early inflammatory biomarkers that could be used to predict or indicate disease progression from simple fatty liver (simple steatosis) to NAFLD/NASH 2) and biomarkers of fibrosis that could be used in the absence of biopsy to stage disease (fibrosis deposition). A cohort of alcoholic liver disease (ALD) subjects were recruited for the pilot study to determine if the biomarker levels identified in the study were indicative (or their trajectory) of worsening liver disease. The pathogenesis of NAFLD and disease progression is complex. Therefore, it is unlikely that single biomarkers would be diagnostic. Therefore, a combination of biomarkers that are diagnostic and/or predictive of NAFLD progression could offer a significant aid in clinical diagnoses and management and treatment of patients with suspected NAFLD.

## Materials and methods

In total, N = 135 patients were recruited for the pilot study (n = 34 (25.2%) controls; n = 26 (19.3%) simple steatosis; n = 61 (45.2%) NAFLD/NASH; and n = 14 (10.4%) ALD). Patients were recruited by Discovery Life Sciences, California, US. Patient samples were de-identified and publicly available and were thus exempt from the requirement of the Institutional Review Board (IRB) approval (Exempt Category 4, IRB/EC). Discovery Life Sciences patient samples were procured pursuant to informed consent provided by the individual under approved protocols 45 CFR 46.116. Serum samples (1 ml) with clinical history was obtained for each subject. Clinical diagnosis of patients and their inclusion in the pilot study was based on ICD-10 coding.

### Clinical factors and behaviours

Patients involved in the pilot study were matched for age, gender, and ethnicity. BMI data was only available for control, NAFLD/NASH and ALD subjects. In addition, the clinical history i.e., medications (e.g., statins, anti-hypertensives etc.) and comorbidities (e.g., hypertension, diabetes etc.), for individuals involved in the study, was limited and data is presented were available.

## Biomarker analysis

### High-sensitivity cytokine array I

Patient samples were analysed in duplicate (n = 2) by Randox Laboratory Clinical Services (RCLS), Antrim, Northern Ireland, United Kingdom by scientists blinded to patient group. In total, 12 biomarkers were investigated by Biochip Array Technology (BAT) (High-Sensitivity Cytokine Array I) (Randox Laboratories Ltd., Crumlin, United Kingdom) using an Evidence Investigator analyser (Randox Laboratories Ltd., Crumlin, United Kingdom), following manufacturer’s instructions. The limits of detection (LOD) for the biomarkers on the biochip were as follows: EGF 2.5 pg/ml, IFNγ 2.1 pg/ml, IL-1α 0.9 pg/ml, IL-1β 1.3 pg/ml, IL-2 4.9 pg/ml, IL-4 3.5 pg/ml, IL-6 0.4 pg/ml, IL-8 2.3 pg/ml, IL-10 1.1 pg/ml, MCP-1 25.5 pg/ml, TNFα 3.7 pg/ml, and VEGF 10.8 pg/ml (McBride et al., 2019; Kurth et al., 2020).

### Albumin, aspartate aminotransferase and alanine aminotransferase

Three biomarkers were analysed on a clinical chemistry Rx Imola analyser (Randox Laboratories Ltd., Crumlin, United Kingdom) according to manufacturer’s instructions: albumin (range 2.87–75.6 g/L), AST (range 4.42–657 U/l) and ALT (range 7–56 U/l).

### ELISAs

Five biomarkers were evaluated using ELISAs, according to manufacturer’s instructions: Midkine (LyraMid, Sydney, Australia), mean detectable dose (MDD) 8 pg/ml, Fatty Acid-Binding Protein-1 (FABP-1) (Abcam, Cambridge, United Kingdom), MDD 1.0 ng/ml, Procollagen-III-peptide (PIIINP) (Cusabio, Houston, US), MDD 0.078 ng/ml, Apolipoprotein F (ApoF) (LSBio, Seattle, United Kingdom), MDD 0.41 ng/ml, and Suppression of Tumorigenicity (ST2/IL-33R) (R&D Systems, Abingdon Science Park, United Kingdom), MDD 5.1 pg/ml. Biomarker results below the LOD or MDD were recorded as 90% of the LOD (Kurth et al., 2020).

### Statistical analysis

Statistical analyses were undertaken using R version 4.0.5. Kruskal-Wallis rank sum test was used to identify significant differences expressed between biomarkers for the four groups (control, simple steatosis, NASH/NAFLD and ALD). The Wilcoxon rank sum test was used to identify which pairs of groups were significantly different. Biomarkers with a *p* < 0.05 were considered significant. Stars of significance are used to indicate *p*-value level; *p < 0.05, **p < 0.01, ***p < 0.001, ****p < 0.0001.

## Results

The clinical characteristics, and groups, for the patients involved in the pilot study are described in Table 1. Patients were matched for age, gender, and ethnicity. BMI data was not available for patients with simple steatosis. No data was available for patient medications and comorbidities.

**TABLE 1:** Patient demographics.

Factor	Control	Simple steatosis	NAFLD/NASH	ALD	*p*-value
Age	54.6 ± 15.3 (n = 34)	53.4 ± 12.5 (n = 26)	51.6 ± 14.1 (n = 61)	50.8 ± 12.7 (n = 14)	0.175
Gender(M)	21/34 (61.8%)	13/26 (50%)	31/61 (50.8%)	9/14 (64.3%)	0.614
Ethnicity (Caucasian)	22/34 (64.7%)	20/25 (80%)	45/58 (77.6%)	10/14 (71.4%)	0.166
BMI	31.9 ± 13.6 (n = 33)	-	34.6 ± 8.6 (n = 40)	32.2 ± 8.5 (n = 5)	0.076

### High-sensitivity cytokine array I

**FIGURE 1:**
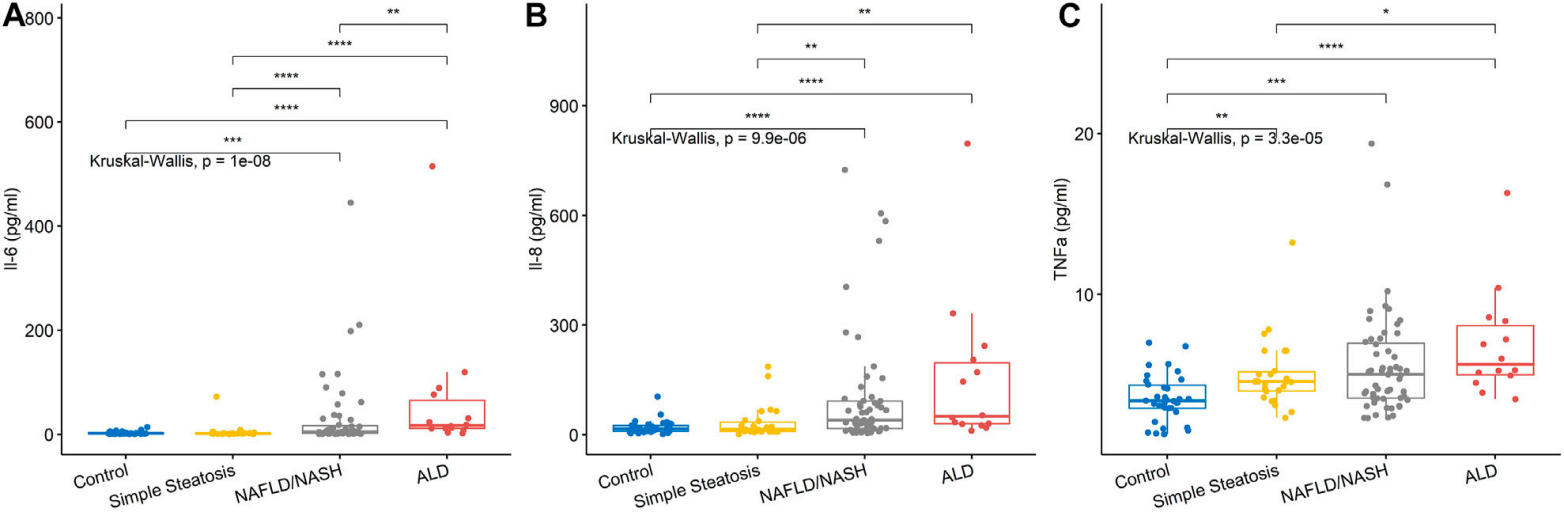
Serum levels for biomarkers of inflammation. **(A)** IL-6 levels and **(B)** IL-8 levels were significantly higher in the NAFLD/NASH and ALD group compared to control and simple steatosis groups. **(C)** TNFα levels were significantly higher in the simple steatosis, NAFLD/NASH, and ALD groups compared to control subjects. Stars of significance; *p < 0.05, **p < 0.01, ***p < 0.001, ****p < 0.0001.

Seven/12 (58.3%) of the biomarkers on the high-sensitivity cytokine array I were significantly different across groups, namely, EGF, INFγ, IL-1β, IL-6, IL-8, IL-10, and TNFα (Table 2 and Figure 1). The biomarker results from the high-sensitivity cytokine array I showed that the cytokine levels overlapped between groups.

**TABLE 2:** High-sensitivity cytokine array I results.

Factor	Control (n = 34)	Simple steatosis (n = 26)	NAFLD/NASH (n = 55)	ALD (n = 14)	*p*-value
EGF (pg/ml)	59.7 ± 62.9	63.8 ± 40.3	46.7 ± 56.0	18.7 ± 21.5	0.010
INFγ (pg/ml)	0.6 ± 1.0	1.3 ± 5.6	0.2 ± 0.3	1.5 ± 3.2	<0.001
IL-1α (pg/ml)	0.2 ± 0.2	0.1 ± 0.1	0.1 ± 0.1	0.2 ± 0.2	0.170
IL-1β (pg/ml)	1.2 ± 1.3	0.7 ± 0.4	2.5 ± 7.7	1.4 ± 1.0	0.015
IL-2 (pg/ml)	1.2 ± 2.0	0.7 ± 1.1	0.7 ± 1.4	1.1 ± 1.2	0.070
IL-4 (pg/ml)	1.6 ± 1.3	1.5 ± 0.8	1.2 ± 0.4	1.3 ± 0.5	0.108
IL-6 (pg/ml)	2.7 ± 2.8	4.7 ± 13.9	31.0 ± 72.5	67.0 ± 133.9	<0.001
IL-8 (pg/ml)	20.2 ± 18.8	33.4 ± 45.3	105.0 ± 162.9	153.1 ± 210.9	<0.001
IL-10 (pg/ml)	0.7 ± 0.7	0.6 ± 0.8	1.1 ± 1.8	3.8 ± 9.3	0.006
MCP-1 (pg/ml)	163.2 ± 90.1	183.7 ± 114.3	224.1 ± 134.0	249.2 ± 223.3	0.183
TNFα (pg/ml)	3.5 ± 1.5	5.0 ± 2.2	5.6 ± 3.2	6.9 ± 3.3	<0.001
VEGF (pg/ml)	103.8 ± 105.4	102.6 ± 70.7	105.5 ± 107.4	102.3 ± 119.1	0.850

### Albumin, aspartate aminotransferase, and alanine aminotransferase

Three biomarkers were evaluated on the Rx Imola analyser (Randox Laboratories Ltd., Crumlin, UK), namely, albumin, AST, and ALT. All three biomarkers were significantly different across groups (Table 3).

**TABLE 3:** Albumin, ALT and AST results.

Factor	Control (n = 34)	Simple steatosis (n = 26)	NAFLD/NASH (n = 61)	ALD (n = 14)	*p*-value
Albumin (g/L)	41.3 ± 4.3	50.4 ± 10.6	38.3 ± 8.5	35.6 ± 8.2	<0.001
AST (U/l)	24.7 ± 16.0	37.3 ± 25.2	80.2 ± 178.0	70.5 ± 62.3	<0.001
ALT (U/l)	23.5 ± 28.2	33.9 ± 33.8	75.9 ± 180.2	24.6 ± 20.5	0.004

### ELISA results

**FIGURE 2:**
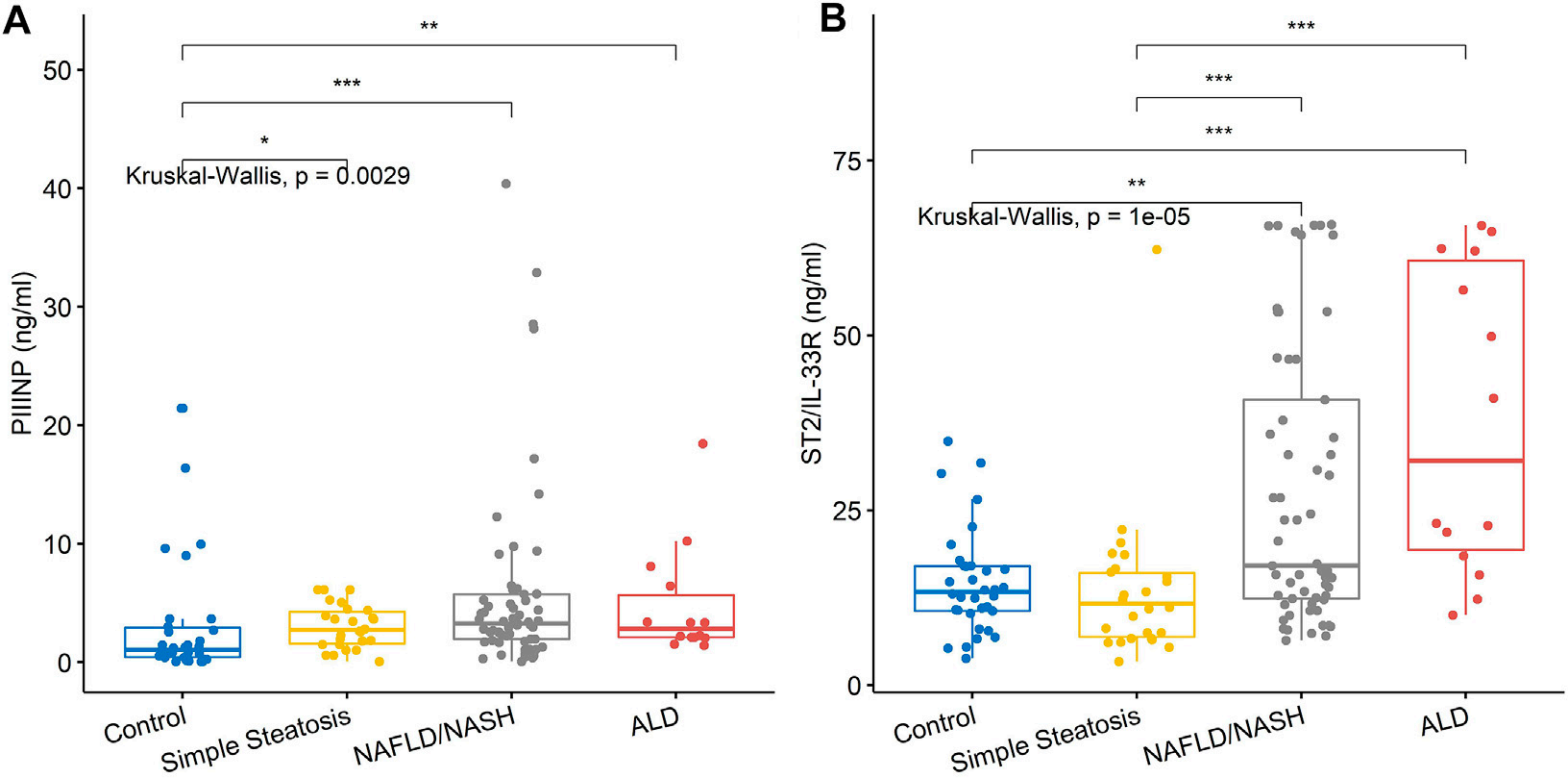
Serum levels for biomarkers of fibrosis. **(A)** PIIINP levels were significantly higher in the NAFLD/NASH and ALD groups compared to simple steatosis and control subjects. **(B)** ST2/IL-33R was significantly higher in the NAFLD/NASH and ALD groups compared to simple steatosis and control subjects. Stars of significance; *p < 0.05, **p < 0.01, ***p < 0.001.

Three/5 (60%) biomarkers measured by ELISA were significantly different across groups, namely, PIIINP, ST2/IL-33R and FABP-1 (Table 4 and Figure 2).

**TABLE 4:** ELISA results.

Factor	Control (n = 34)	Simple steatosis (n = 26)	NAFLD/NASH (n = 61)	ALD (n = 14)	*p*-value
PIIINP (ng/ml)	3.5 ± 5.8	3.0 ± 1.8	5.8 ± 7.9	4.8 ± 4.7	0.003
MK (pg/ml)	720.1 ± 1130	571.7 ± 797.8	941 ± 1401	942.8 ± 1190.6	0.658
ST2/IL-33R (ng/ml)	14.7 ± 7.4	13.5 ± 11.2	27.7 ± 19.9	37.6 ± 21.8	<0.001
FABP-1 (ng/ml)	52.0 ± 56.6	34.3 ± 43.7	44.9 ± 42.3	94.9 ± 67.6	0.007
ApoF (µg/ml)	7.7 ± 9.7	5.8 ± 3.7	4.9 ± 3.7	6.5 ± 5.8	0.385

## Discussion

Non-alcoholic fatty liver disease is a silent disease with few or no symptoms (Newton, 2010). However, certain health conditions, including obesity, metabolic syndrome, and type-2 diabetes make individuals more likely to develop the disease (National Guideline Centre (UK), 2016a). Having high levels of fat in the liver are associated with an increased risk of heart attacks and strokes (Fudim et al., 2021; Xu et al., 2021). Currently, no medicines have been approved to treat NAFLD or NASH (Mishra and Younossi, 2007). However, if the disease is detected and managed at an early stage, it’s possible to stop NAFLD getting worse and reduce the amount of fat in the liver (National Guideline Centre (UK), 2016b).

The liver normally contains some fat however, an individual is considered to have a fatty liver (hepatic steatosis) if the liver contains more than 5%–10% fat. Fat deposition in the liver may cause increased levels of liver enzymes that are detected during routine blood tests (e.g., AST and ALT). ALT is a liver enzyme that converts proteins into energy for liver cells. AST helps metabolise amino acids. ALT and AST are normally present in blood at low levels. An increase in ALT and/or AST may indicate liver damage, disease or muscle damage (Sanyal et al., 2015).

Ten to 30% of individuals with NAFLD will develop inflammation of the liver (non-alcoholic steatohepatitis (NASH)) (Neuman et al., 2014). Mild damage to the liver is reversible however, severe, or long-term damage can result in fibrosis, resulting in irreversible liver disease (cirrhosis) (Jung and Yim, 2017). Cirrhosis, liver cancer, and liver failure are serious conditions that are life-threatening (Wree et al., 2013). Unfortunately, once this stage of liver disease has been reached, treatment options are limited. Therefore, it is important to catch liver disease early, in the inflammation and fibrosis states.

To date, a limited number of biomarkers have been identified to triage patients with liver disease (Wong et al., 2018; De and Duseja, 2020; Schuppan et al., 2022). Normal levels of biomarkers can vary considerably in healthy individuals based on gender, ethnicity, age, nutritional status, comorbidities, and potential synergistic effects of medications. As yet, no single biomarker, or combinations thereof, have the sensitivity or specificity to replace the gold standard, liver biopsy (Tincopa, 2020).

In this pilot study, we compared serum biomarkers of liver disease, inflammation, and fibrosis in control individuals to patients clinically diagnosed with either simple steatosis, NAFLD/NASH or ALD. Unsurprisingly, patients with a diagnosis of simple steatosis and/or NAFLD/NASH had significantly elevated serum levels of AST and ALT when compared to control participants. Furthermore, lower serum albumin was detected for the NAFLD/NASH and ALD patients, consistent with abnormalities in liver function.

Inflammatory biomarkers IL-1β, IL-6, IL-8, IL-10, and TNFα were all significantly elevated in the NAFLD/NASH patients, with respect to control participants and patients with simple steatosis. TNFα was significantly higher in the simple steatosis patients with respect to control individuals. However, TNFα was not significantly different between simple steatosis and NAFLD/NASH patients, suggesting that TNFα may be an acute phase biomarker that is elevated in early liver disease.

PIIINP and ST2/IL-33R, biomarkers of liver fibrosis, were elevated only in the NAFLD/NASH and ALD patients, suggesting that both biomarkers detect fibrosis and late-stage liver disease. However, of interest was the distribution of the serum ST2/IL-33R results observed for the NAFLD/NASH and ALD patients. There was no significant difference between the NAFLD/NASH group and ALD patients. Unfortunately, as we did not have access to patient tissue biopsy results, we could not correlate the fibrotic stage of the tissue with serum ST2/IL-33R levels. However, we hypothesised that the distribution of serum ST2/IL-33R levels for NAFLD/NASH patients may be related to stage and grade of their liver disease. However, this hypothesis would need verification in NAFLD/NASH patients where a blood and tissue biopsy (pathologically proven) sample were available for each patient.

Our data would suggest that TNFα may be a useful early biomarker for differentiating control participants from patients with early-stage liver disease (simple steatosis). Furthermore, serum ST2/IL-33R levels may give an indication of the extent of liver disease (NAFLD/NASH). However, using a combination of biomarkers, e.g., TNFα and ST2/IL-33R, may allow risk stratification of patients who present with mild liver disease and who are averse to biopsy. Combining biomarker results with clinical risks may allow clinicians to manage their patients based on biomarker results (depending on cut-off values) and clinical risk scores (e.g., BMI, comorbidities etc.). Patients that are positive for both biomarker and clinical risk score would be designated as ‘high-risk’ for liver disease progression. Thus, patients identified as ‘high-risk’ would be triaged for biopsy. Whereas patients identified as ‘low-risk’ of disease progression, could be monitored and managed in primary care.

## Conclusion

In conclusion, our data suggests that TNFα may be a useful biomarker for staging patients with early liver disease (simple steatosis), when considered with known clinical risks for NAFLD e.g., BMI, triglycerides, LDL, HDL, medications (e.g., statins, antihypertensives), and co-morbidities (i.e., diabetes, CVD). Furthermore, serum ST2/IL-33R levels may correlate with the extent of liver fibrosis deposition and disease progression. Patients that are averse to biopsy could potential be stratified into ‘low’ and ‘high’ risk of disease progression using the biomarker combination described. However, a longitudinal study, where liver biopsy and blood samples are available, including a detailed patient clinical history, is warranted to confirm our findings.

### Clinical utility of the biomarkers

To test if a biomarker will add to risk prediction is based on (a) model discrimination, (b) model calibration, and (c) risk reclassification. Therefore, addition, of the biomarkers TNFα and ST2/IL-33R, to know risk factors for NAFLD/NASH, would allow clinicians to both identify patients at potential risk of disease progression and monitor any therapeutic intervention.

### Limitations of the study

This is a pilot study and as such there are several limitations that must be considered: the patient sample volume that was available was limited and therefore a subset of biomarkers, selected by the authors, were investigated; the total number of patients in each of the subgroups was limited; patients were clinically diagnosed in the absence of a liver biopsy and thus there is the potential risk of under- and over-reporting a clinically incorrect diagnosis. Clinical information and history on individual patients were limited, and in some instances absent e.g., medications, comorbidities, BMI.

## Data Availability

The raw data supporting the conclusion of this article will be made available by the authors, without undue reservation.
